# Dietary supplementation with walnut (*Juglans regia* L.) green husk polyphenol extract mitigates fatty liver hemorrhagic syndrome in laying hens

**DOI:** 10.3389/fvets.2026.1803328

**Published:** 2026-06-12

**Authors:** Jinyan Yun, Xuezhao Sun, Caoxing Huang, Wei Zhang, Ziji Wang, Linfeng Zhang, Zhenfeng Li, Jiayin Sun, Baishuang Yin

**Affiliations:** 1 College of Animal Science and Technology, JiLin Agricultural Science and Technology College, Jilin, Jilin Province, China; 2 Co-Innovation Center for Efficient Processing and Utilization of Forest Resources, Department of Bioengineering, Nanjing Forestry University, Nanjing, Jiangsu Province, China; 3 College of Traditional Chinese Medicine, JiLin Agricultural Science and Technology College, Jilin, Jilin Province, China

**Keywords:** antioxidation, cecal microbiota, fatty liver hemorrhagic syndrome, gut microbiota, laying hen, oxidative stress, walnut green husk polyphenol extract

## Abstract

**Background:**

Extracts derived from the walnut (*Juglans regia* L.) green husk exhibit a variety of biological activities. This study investigated the effects of walnut green husk polyphenol extracts (WGHPE) on fatty liver hemorrhagic syndrome (FLHS)-related indicators, antioxidant performance, and cecal microbiota modulation in laying hens.

**Methods:**

A total of 350 Hy-Line Brown laying hens aged 43 weeks were randomly assigned to five groups with seven replicates per group and 10 hens per replicate. An FLHS model was induced via intramuscular injection of *β*-estradiol dissolved in corn oil. The control (Con) and FLHS model groups received a basal diet, whereas three FLHS-based treatment groups were fed the basal diet supplemented with 0.5% (WGHPEL), 1.0% (WGHPEM), or 1.5% (WGHPEH) WGHPE, respectively. All laying hens had unrestricted access to food and water throughout the 8-week experimental period.

**Results:**

Compared with the FLHS group, dietary supplementation with WGHPE significantly reduced liver weight, liver coefficient, abdominal adipose weight, and abdominal adipose coefficient. Histological evaluation demonstrated that WGHPE alleviated hepatocellular vacuolar degeneration and lipid droplet accumulation, indicating an improvement in FLHS-related pathological features. Furthermore, WGHPE significantly reversed FLHS-induced elevations in serum levels of total cholesterol, aspartate aminotransferase, alanine aminotransferase, and low-density lipoprotein cholesterol. WGHPE also enhanced systemic antioxidant capacity by increasing catalase and total superoxide dismutase activities in *β*-estradiol/corn oil-induced laying hens, while decreasing malondialdehyde levels. Regarding intestinal health, WGHPE significantly increased villus height and the villus-to-crypt ratio in the jejunum and ileum. Furthermore, in the WGHPE treatment group, the relative abundance of beneficial bacterial taxa was increased. *Campylobacter* and *Parasutterella* were positively correlated with body weight and abdominal adipose deposition, whereas *Desulfovibrio* and *unclassified_Oscillospiraceae* showed negative correlations. These findings collectively indicate beneficial associations between dietary WGHPE supplementation, intestinal microbiota composition, and overall health status in laying hens with FLHS.

**Conclusion:**

Dietary supplementation with WGHPE mitigated *β*-estradiol/corn oil-induced FLHS-associated liver injury, enhanced antioxidant capacity, and improved intestinal morphology and microbial composition. A supplementation level of 1.5% WGHPE is recommended for optimal efficacy.

## 1 Introduction

In recent years, the rapid development of the poultry farming industry has been accompanied by substantial changes to nutritional strategies, increased stocking densities, and restricted physical activity. Consequently, laying hens are increasingly exposed to multiple metabolic and environmental stressors during production. Among these challenges, fatty liver hemorrhagic syndrome (FLHS) represents one of the most prevalent and economically damaging nutritional metabolic diseases affecting laying hens (1). The pathological characteristics of FLHS include rapid body weight gain, yellowish liver discoloration, liver enlargement with a soft and fragile texture, pronounced hemorrhagic spots on the liver surface, excessive abdominal fat deposition, and, in severe cases, liver rupture leading to internal hemorrhage and mortality (2). FLHS reportedly accounts for approximately 40–70% of mortality in affected laying hen flocks, resulting in significant economic losses for poultry producers (3).

Fatty liver hemorrhagic syndrome arises from dysregulated lipid metabolism and oxidative stress driven by nutritional, environmental, hormonal, genetic, metabolic, and gut microbial factors (4), a process often termed the “multiple parallel hits” hypothesis (5). However, the precise pathogenesis remains unclear, and effective clinical treatments are limited. Therefore, identifying effective approaches to alleviate FLHS-associated liver injury is critical to maintaining stable production performance and egg quality (6).

The exocarp of walnut, commonly referred to as the green husk, is a by-product generated during the processing of fresh walnuts and is often discarded as waste (7), leading to environmental pollution and the underutilization of biological resources. Walnut green husk contains a wealth of active components, including phenols, quinones, polysaccharides, and flavonoids (8, 9). Notably, its polyphenolic extract exhibits multiple pharmacological properties, such as antibacterial activity (10, 11), immune modulation (12, 13), antioxidant effects (14), growth promotion capabilities (15), and enhancement of intestinal microecological balance (16–18). Representative plant polyphenols include quercetin, hesperetin, caffeic acid, chlorogenic acid, ferulic acid, gallic acid (as part of phenolic acids), and resveratrol (a stilbene) (19, 20). Existing studies on walnut green husk polyphenol extract (WGHPE) have primarily focused on broiler growth performance (21), feed conversion efficiency (22, 23), and laying hen production traits (24, 25).

While our previous research indicates that WGHPE supplementation is safe for laying performance and egg quality and may exert beneficial effects on immune function and intestinal morphology, its role in FLHS-associated liver injury has not yet been investigated (26). To further investigate the potential of WGHPE in mitigating liver damage in FLHS laying hens, we hypothesized that its dietary inclusion could alleviate hepatic injury by modulating oxidative stress and gut microbiota homeostasis. To test this hypothesis, an FLHS animal model combined with gut microbial diversity sequencing was employed (27). The objective was to elucidate the correlations between the gut microbiota community and key physiological indicators, including antioxidant parameters. Collectively, this study provides new insights into the development of green feed additives for laying hens and supports the valorization of natural plant resources.

## 2 Materials and methods

### 2.1 Ethics

All experimental procedures involving animals were approved by the Animal Ethics and Welfare Committee of JiLin Agricultural Science and Technology College, Jilin City, Jilin Province, China (Approval No. 20231015) and were conducted in accordance with the institutional animal welfare guidelines. The study was carried out at the Animal Experimental Station of the same institution.

### 2.2 Preparation of WGHPE

The preparation of the polyphenol extracts from the walnut green husk was conducted following the method described by Cosmulescu et al. (28), with some modifications. In mid to late July and early August, green husks of wild *Juglans regia L.* were collected from the Changbai Mountains in China. The collected materials, which weighed 20 kg, were air-dried until a constant weight was achieved and subsequently ground into a coarse powder. A 70% ethanol solution served as the extraction solvent at a solid-to-liquid ratio of 1:10 (w/v). The extraction process was performed twice for 1.5 h each time under reflux conditions. The combined extracts were filtered through absorbent defatted cotton, and the filtrate was concentrated under reduced pressure until the odor of ethanol disappeared. The residue was reconstituted with distilled water to a final volume of 60 L. The resulting solution was allowed to stand at room temperature for 12 h to facilitate sedimentation, followed by filtration through absorbent defatted cotton to remove precipitated material. The total phenolic content of the final extract was measured at 3.2 mg/mL (expressed as gallic acid equivalents). The measurement methods for the compounds are detailed in the Supplementary materials, with further information provided in Supplementary Table 1.

### 2.3 Experimental design and management

After a one-week adaptation period, 350 Hy-Line Brown laying hens, aged 43 weeks and with similar body weights (1.60 ± 0.2 kg), were randomly assigned to five groups (seven replicates per group, 10 hens per replicate). Except for the control group (Con), hens in the remaining four groups were subjected to FLHS induction via intramuscular injection of *β*-estradiol (7.5 mg dissolved in 0.5 mL corn oil per kg body weight) every 4 days for a total of three injections. Based on previous findings indicating improved immune function without adverse effects on laying performance or egg quality, 0.5, 1.0, and 1.5% (v/w, extract volume per diet mass) were chosen as the low, medium, and high doses, respectively, for the subsequent 8-week experiment. Briefly, the induced FLHS model was further divided into the following treatment groups: the FLHS model group (FLHS) receiving a basal diet; the low-dose WGHPE treatment group (WGHPEL) receiving a basal diet supplemented with 0.5% WGHPE; the medium-dose WGHPE treatment group (WGHPEM) receiving a basal diet supplemented with 1.0% WGHPE; and the high-dose WGHPE treatment group (WGHPEH) receiving a basal diet supplemented with 1.5% WGHPE. The dietary inclusion levels of WGHPE (0.5, 1.0, and 1.5%) were calculated based on the volume-to-mass ratio of the liquid extract to the basal diet. The basal diet (composition and nutritional levels shown in Supplementary Table 2 were prepared in strict accordance with the nutritional recommendations provided in the Hy-Line Brown Commercial Layers Management Guide.^1^ Throughout the 8-week experimental period, all laying hens had unrestricted access to feed and water, while chicken houses were cleaned and disinfected weekly to ensure a clean and hygienic environment.

This study was part of a larger project. The other part of the project about the effects of WGHPE on laying performance, egg quality, and immunity was reported earlier (26).

### 2.4 Sample collection

At the conclusion of the experiment, two laying hens with a body weight close to the group mean were selected from each replicate. Following a 12-h fasting period, venous blood was collected, and serum was separated. The blood was allowed to stand at room temperature for 2 h and then centrifuged at 3000 × *g* for 10 min at 4 °C. The resulting supernatant was stored at −80 °C for subsequent analysis. Both hens from each replicate were used for serum biochemical and histological analyses (liver and intestine), and all measurements were based on independent biological replicates (*n* = 14 per group for each assay). Following blood collection, the laying hens were anesthetized with isoflurane (1.5–2% in oxygen) and euthanized by intravenous injection of sodium pentobarbital (≥120 mg/kg body weight). After euthanasia, the entire liver and abdominal adipose tissue were excised and weighed. Liver coefficient (relative liver weight) was calculated as: liver weight/body weight × 100. The liver and middle sections of the duodenum, jejunum, and ileum underwent gentle rinsing with physiological saline and were fixed in 4% paraformaldehyde for further histological examination. From each replicate, one of the two hens was randomly selected for cecal content collection. The samples were snap-frozen in liquid nitrogen and stored at −80 °C for gut microbiota analysis, yielding *n* = 7 per group.

### 2.5 Serum biochemical and antioxidant activities

The levels of serum biochemical indicators, including triglyceride (TG), total cholesterol (TC), high-density lipoprotein cholesterol (HDL-C), low-density lipoprotein cholesterol (LDL-C), alanine aminotransferase (ALT), and aspartate aminotransferase (AST), as well as antioxidant indicators such as malondialdehyde (MDA), total superoxide dismutase (T-SOD), glutathione peroxidase (GSH-Px), and catalase (CAT) activity in serum were measured using an Absorbance Microplate Reader (VersaMax, Molecular Devices, United States). All detection kits were procured from Nanjing Jiancheng Bioengineering Institute, Nanjing, China.

### 2.6 Morphological examination of liver and intestine

The duodenum, jejunum, and ileum samples were fixed in 4% paraformaldehyde, followed by dehydration and embedding in paraffin. The sections were subsequently stained with hematoxylin and eosin (H&E). Imaging of the small intestinal tissue was performed at magnifications of 40× and 100×, respectively, using an Amex1000 imaging system (Life Technologies Inc., United States). Measurements of villus height, crypt depth, and the villus height/crypt depth (V/C) ratio for the intestinal tissue were conducted using Image-Pro Plus 6.0 software. Liver samples were stained with H&E using the same method as above. Pathological changes in the liver tissue, including degeneration, inflammation, edema, and congestion, were evaluated and described. The fixed liver tissues were embedded in optimal cutting temperature (OCT) compound and sectioned utilizing a cryostat. Following rapid freezing, liver sections were stained with Oil Red O at a magnification of 400 × to assess lipid droplet accumulation. Data visualization was performed in GraphPad Prism 9.5 (GraphPad Software Inc., San Diego, CA, United States).

### 2.7 Cecal microbiota analysis

Total genomic DNA was extracted from cecal content using the TGuide S96 Magnetic Soil/Stool DNA Kit (Tiangen Biotech Co., Ltd.) according to the manufacturer’s instructions. The hypervariable region V3-V4 of the bacterial 16S rRNA gene was amplified with primer pairs 338F: 5′- ACTCCTACGGGAGGCAGCA-3′ and 806R: 5′- GGACTACHVGGGTWTCTAAT-3′. The amplified products were purified with the Omega DNA purification kit (Omega Inc., Norcross, GA, United States) and quantified using Qsep-400 (BiOptic, Inc., New Taipei, China). The amplicon library was paired-end sequenced (2 × 250) on an Illumina Novaseq 6,000 (Beijing Biomarker Technologies Co., Ltd., Beijing, China).

The raw image data files obtained from high-throughput sequencing were converted into raw sequence reads through base calling analysis and stored in FASTQ file format, which contains sequence reads and corresponding quality information. In the quality filtering process, sequences with similarity > 97% were clustered into the same operational taxonomic unit (OTU) by USEARCH (29) (v10), and the OTU counts of less than 2 in all samples were filtered. Taxonomy annotation of the OTUs was performed based on the Naive Bayes classifier in QIIME2 (30) using the SILVA database (31) (release 138.1) with a confidence threshold of 70%. Alpha diversity analysis was performed to identify the complexity of species diversity of each sample utilizing QIIME2 software. Beta diversity calculations were analyzed by principal coordinate analysis (PCoA) to assess the diversity in samples for species complexity. One-way analysis of variance was used to compare bacterial abundance and diversity. The online platform BMKCloud^2^ was used to analyze the sequencing data. After normalizing the OTUs according to the predicted 16S copy number, function prediction and microbial phenotypes were assessed using PICRUST2(v2.3.0), BugBase (version 0.1.0) and FAPROTAX (1.2.6) (32), respectively. The correlation between species in each sample was calculated based on their abundance and variation at the genus level utilizing the Spearman algorithm, with subsequent correlation analysis performed in R version 3.6.1 (packages: psych v2.1.9, igraph v1.2.5, visNetwork v2.1.0). Additionally, using Python version 2.7.8 (scipy version 0.14.1), *p*-values for the correlations of various physiological indicators were computed from the correlation coefficients, and these results were visualized as a heatmap representation of correlations (Heatmap).

### 2.8 Statistical analysis

All data were analyzed using GraphPad Prism version 9.5 (GraphPad Software, San Diego, CA, United States). Results are expressed as replicate (pen) means ± standard error of the mean (SEM), with *n* = 7 independent replicates per treatment group (each replicate consisting of the mean value of two birds sampled from that pen).

The experimental unit was the replicate pen. For comparisons involving only two groups (Control vs. FLHS model), data were analyzed using an unpaired two-tailed Student’s t-test when normally distributed or the Mann–Whitney U test when the normality assumption was violated (Shapiro–Wilk test). For datasets involving more than two treatment groups (Control, FLHS model, WGHPEL, WGHPEM, and WGHPEH), one-way analysis of variance (ANOVA) was performed on replicate means. When the ANOVA F-test was significant (*p* < 0.05), means were separated using the Least Significant Difference (LSD) *post-hoc* test.

Normality of residuals was assessed with the Shapiro–Wilk test and homogeneity of variances with Levene’s test. When either assumption was violated, the non-parametric Kruskal–Wallis test followed by Dunn’s multiple-comparisons test was used instead of ANOVA/LSD. For cecal microbiota data, the same framework (one-way ANOVA or Kruskal–Wallis on replicate means) was applied to *α*-diversity indices, relative abundances at phylum and genus levels, and predicted functional profiles (BugBase and FAPROTAX). Beta-diversity was visualized by partial least squares discriminant analysis (PLS-DA); differences in community structure were tested by PERMANOVA on Bray–Curtis distances (implemented in QIIME2).

Spearman correlation coefficients between genus-level relative abundances and physiological/antioxidant/histological parameters were calculated in R (v3.6.1). A *p*-value < 0.05 was considered statistically significant. All statistical tests were two-sided.

## 3 Results

### 3.1 Liver characteristics of FLHS hens

**Figure 1:**
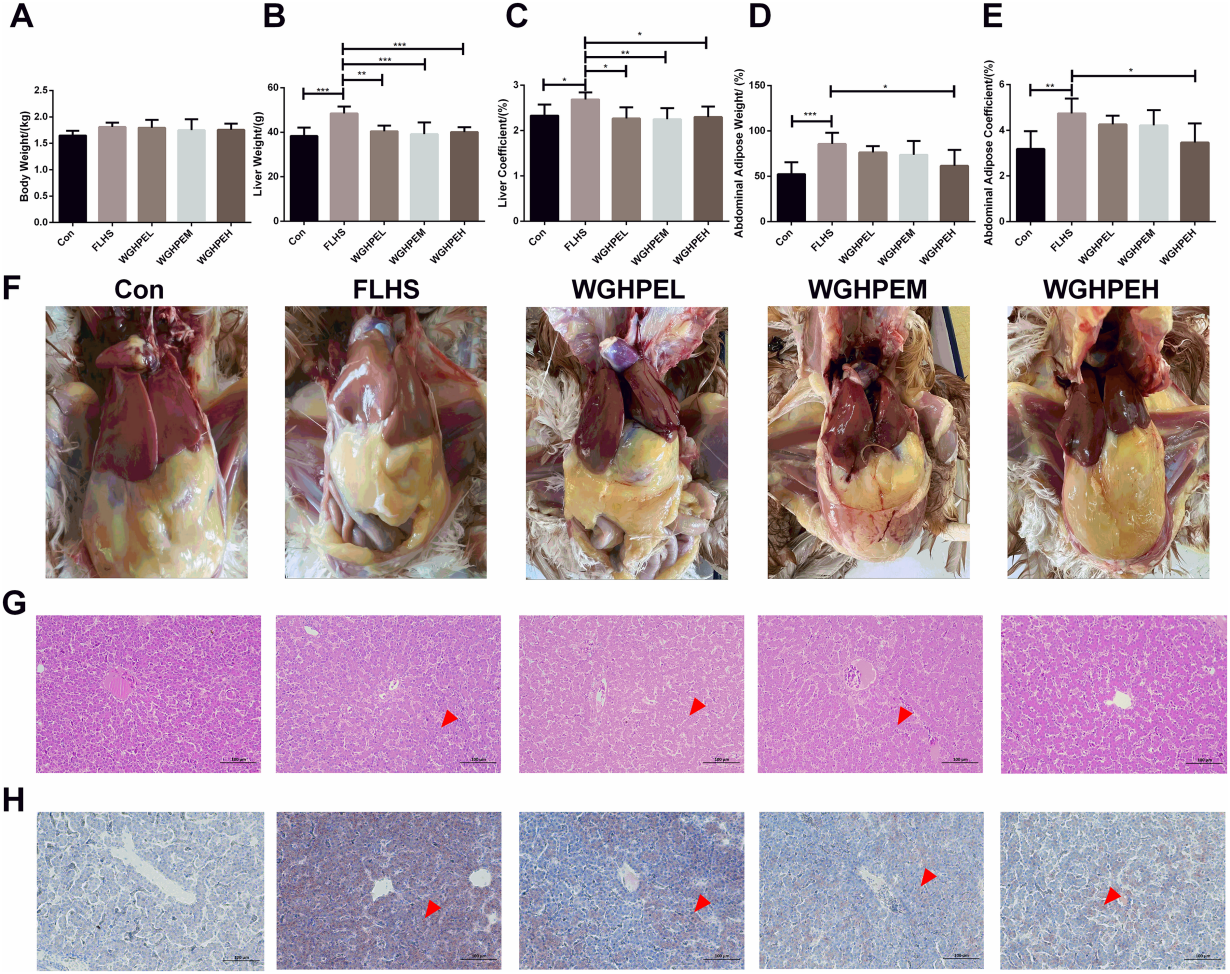
Walnut green husk polyphenol extracts (WGHPE) relieved the symptoms of fatty liver hemorrhagic syndrome (FLHS) and the structural damage in the liver of laying hens. **(A)** Body weight (kg). **(B)** Liver weight (g). **(C)** Liver coefficient (%). **(D)** Abdominal adipose weight (%). **(E)** Abdominal adipose coefficient (%). **(F)** Gross appearance of liver morphology. **(G)** The representative photomicrographs of liver morphology with H&E staining. **(H)** The representative liver sections stained with Oil Red O. Red arrows indicate representative vacuolar degeneration and lipid droplet accumulation. Data are presented as the mean ± SEM; *n* = 7 hens per group. Statistical analysis was performed using one-way ANOVA followed by the LSD. *p < 0.05, **p < 0.01, ***p < 0.001.

Compared to the control group (Con), liver weight, liver coefficient, abdominal adipose weight, and abdominal adipose coefficient in the FLHS group were significantly elevated (*p* < 0.05). However, when compared with the FLHS group, WGHPE supplementation resulted in significant reductions in these indicators in a dose-dependent manner, while body weight remained unaffected (*p* > 0.05; Figures 1A–E). Autopsy findings revealed that livers from hens in the FLHS group exhibited a yellowish-brown coloration and an oily texture, accompanied by increased volume and blunt edges, along with streaky hemorrhagic spots on their surface characteristics closely resembling naturally occurring FLHS (33) (Figure 1F). In contrast, following WGHPEL treatment, no significant improvements relative to the FLHS group were observed; however, high-dose treatment led to marked alleviation of typical symptoms associated with FLHS.

Liver tissues were subjected to H&E staining and Oil Red O staining. The results of the H&E staining revealed that the livers of hens in the FLHS group exhibited severe vacuolar degeneration, characterized by indistinct cell boundaries. In contrast, fat degeneration and vacuolar changes in liver cells were partially alleviated in the WGHPE-treated groups (Figure 1G). Furthermore, findings from Oil Red O staining indicated that in the FLHS group, a significant accumulation of lipid droplets was noted within the cytoplasm of hepatocytes, displacing nuclei towards the periphery and resulting in a scarcity of intact cells. However, both WGHPEM and WGHPEH groups demonstrated a reduction in lipid droplet accumulation within hepatic tissues (Figure 1H).

### 3.2 Lipid metabolism indices

**Figure 2:**
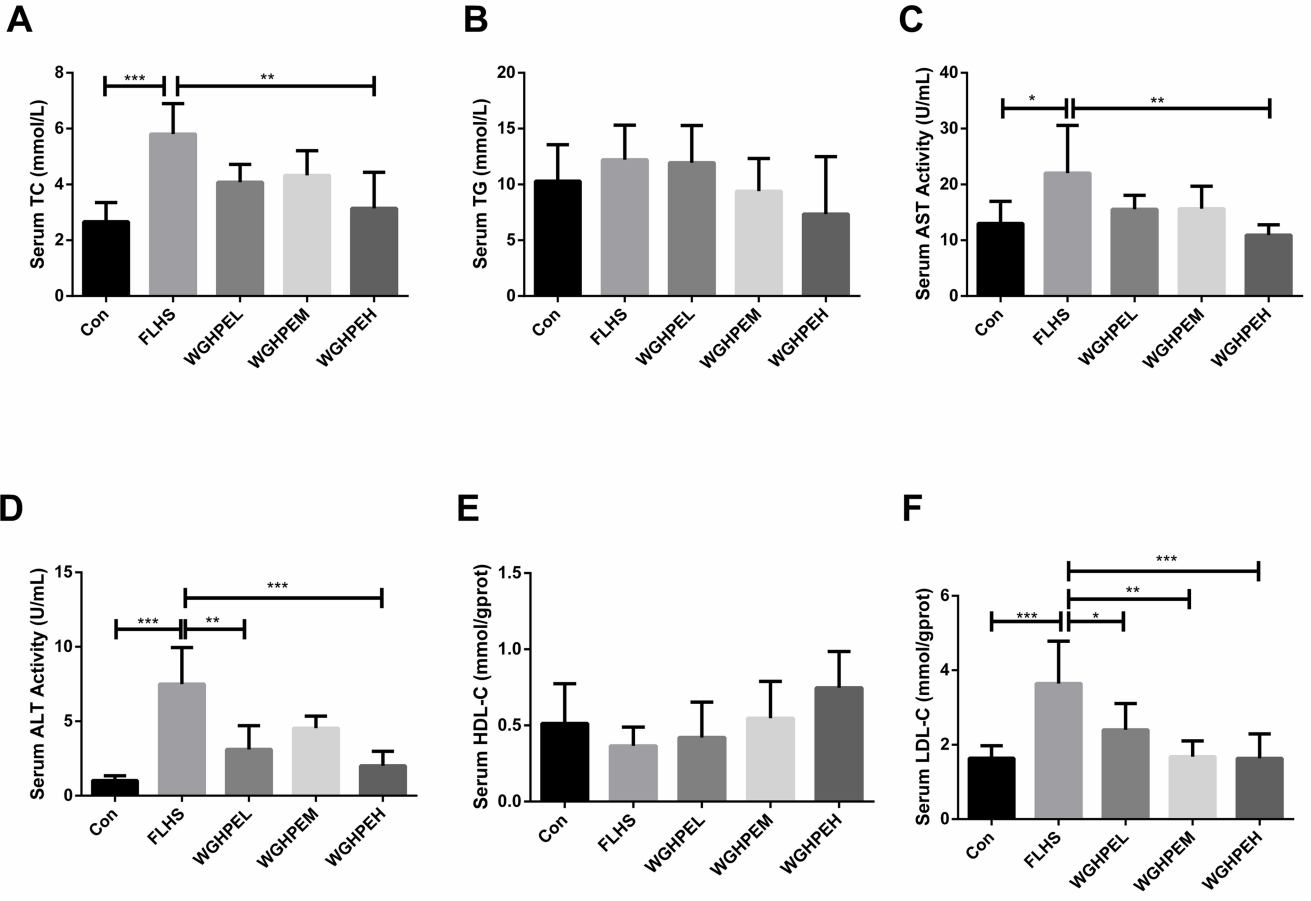
Effects of walnut green husk polyphenol extracts (WGHPE) on serum biochemical indices of fatty liver hemorrhagic syndrome (FLHS) laying hens. **(A)** TC, total cholesterol. **(B)** TG, triglyceride. **(C)** AST, aspartate aminotransferase. **(D)** ALT, alanine aminotransferase. **(E)** HDL-C, high-density lipoprotein cholesterol. **(F)** LDL-C, low-density lipoprotein cholesterol. Data are presented as the mean ± SEM; *n* = 7 hens per group. Statistical analysis was performed using one-way ANOVA followed by the LSD. *p < 0.05, **p < 0.01, ***p < 0.001.

Compared to the FLHS group, the serum levels of TC (*p* < 0.01), AST (*p* < 0.01), ALT (*p* < 0.001), and LDL-C (*p* < 0.001) were significantly reduced following treatment with WGHPEH (Figure 2). Although no statistically significant differences were noted in serum AST levels in the WGHPEL and WGHPEM treatment groups compared to those in the FLHS model group, there remained a non-significant downward trend in the activities of serum ALT and AST within the WGHPEH treatment cohorts, suggesting a potential protective effect exerted by WGHPE on liver function.

### 3.3 Serum antioxidant activity

**Figure 3:**
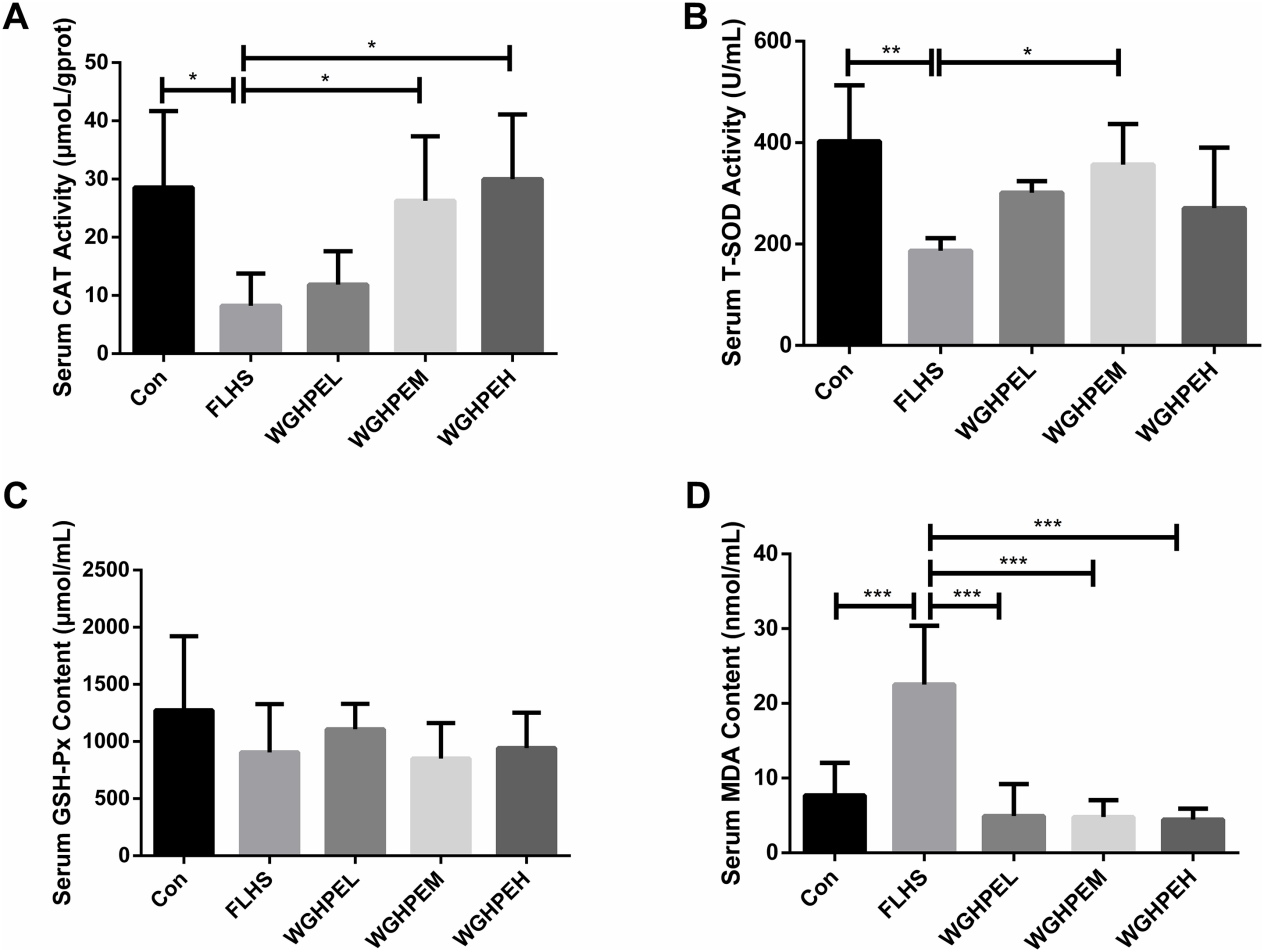
Effects of walnut green husk polyphenol extracts (WGHPE) on serum antioxidant activity of fatty liver hemorrhagic syndrome (FLHS) laying hens. **(A)** CAT, catalase. **(B)** T-SOD, total superoxide dismutase. **(C)** GSH-Px, glutathione peroxidase. **(D)** MDA, malondialdehyde. Data are presented as the mean ± SEM; *n* = 7 hens per group. Statistical analysis was performed using one-way ANOVA followed by the LSD. *p < 0.05, **p< 0.01, ***p < 0.001.

Compared to the control group, serum CAT and T-SOD activities in the FLHS model group were significantly reduced (*p* < 0.05), while MDA levels were significantly elevated by approximately two-fold (*p* < 0.05; Figure 3). Supplementation with WGHPE induced a dose-dependent increase in both CAT and T-SOD levels in the serum of FLHS laying hens (*p* < 0.05), while significantly reducing serum MDA levels (*p* < 0.05). However, serum glutathione peroxidase (GSH-Px) activity did not differ significantly among the five groups (*p* > 0.05; Figure 3C).

### 3.4 Intestinal morphology

**Figure 4:**
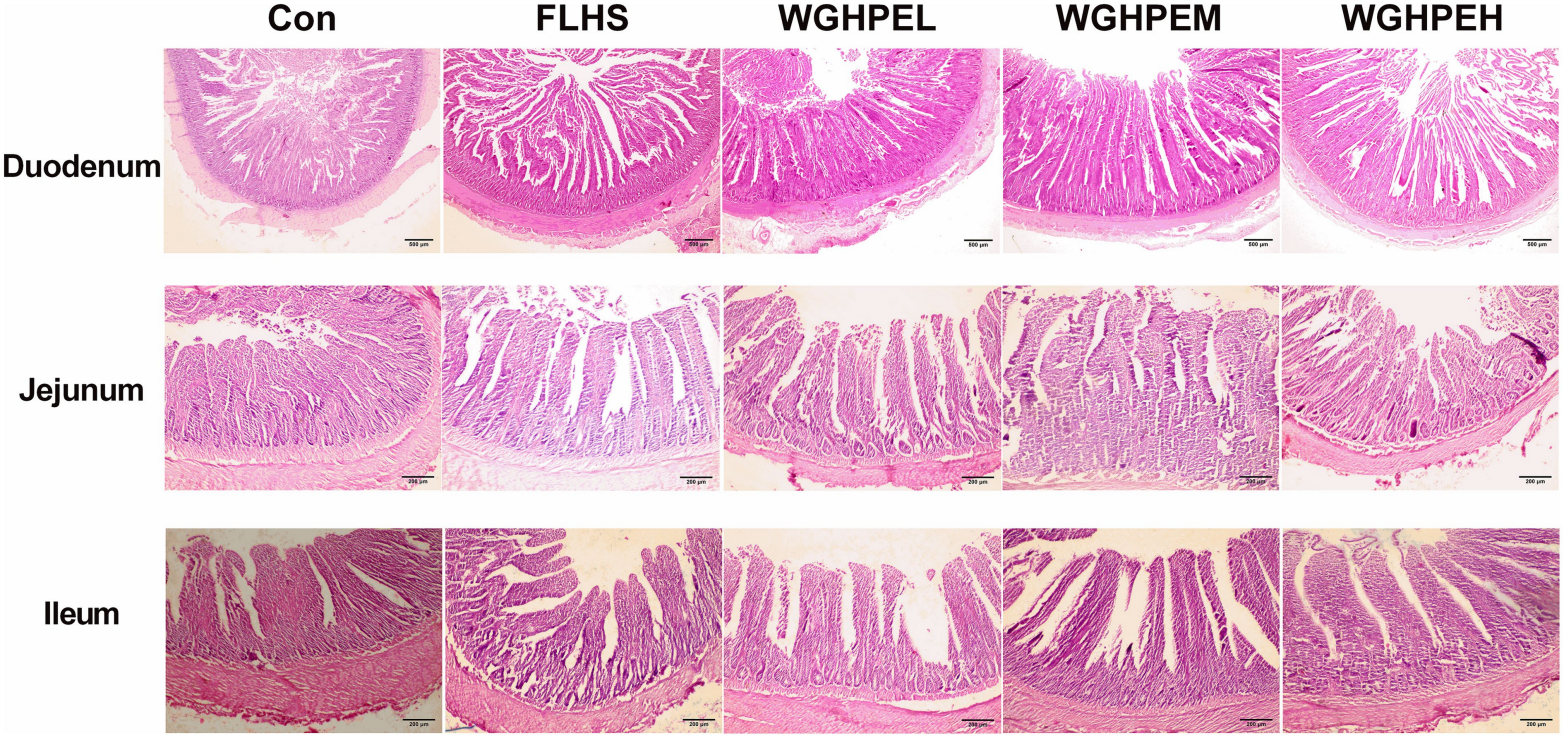
Walnut green husk polyphenol extracts (WGHPE) improved the intestinal morphology of fatty liver hemorrhagic syndrome (FLHS) hens. The representative histological changes of the duodenum, jejunum, and ileum villus height and crypt depth with H&E staining (scale bar = 500 μm). Duodenum 40×; jejunum, ileum 100 × .

**Figure 5:**
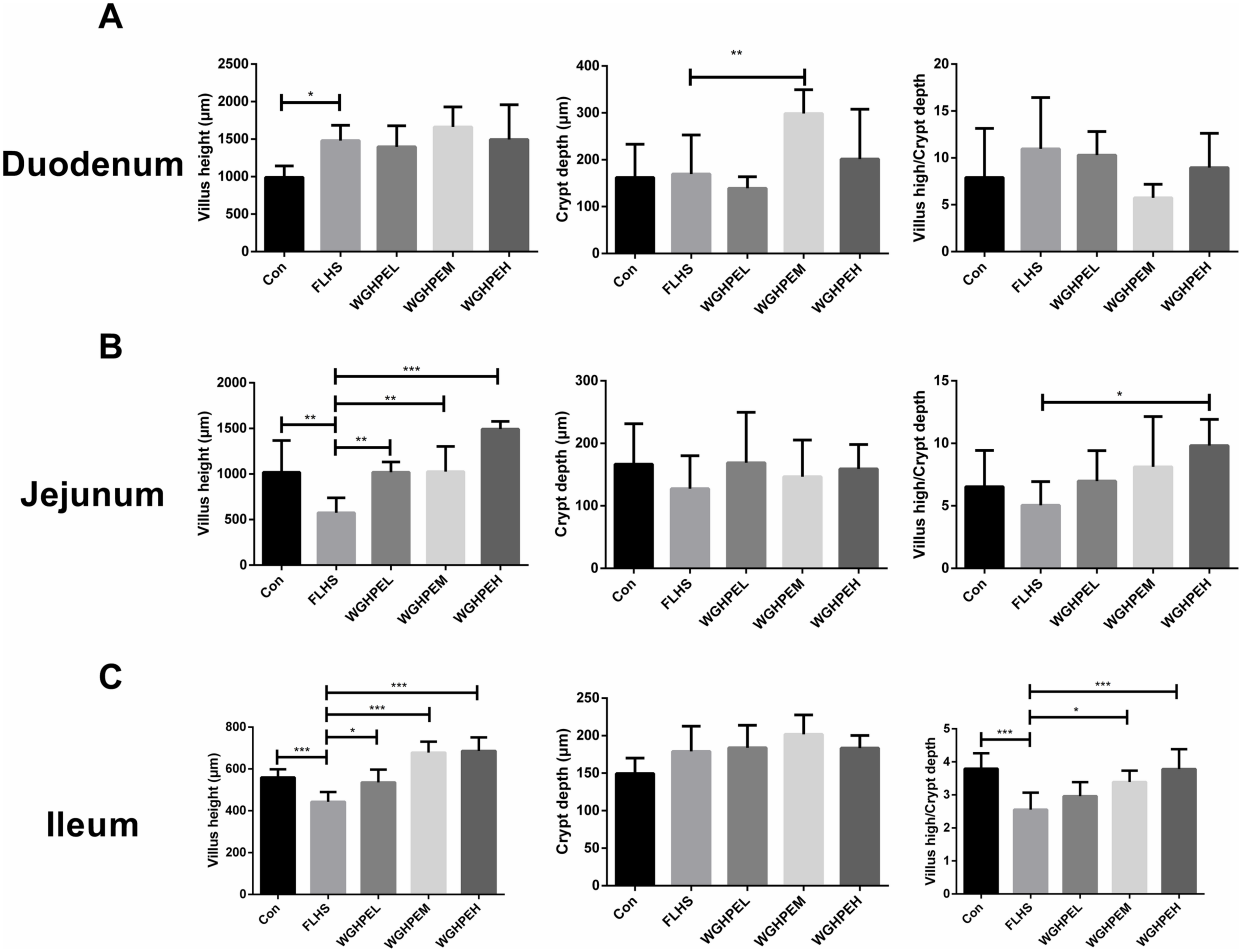
Effects of walnut green husk polyphenol extracts (WGHPE) on villus height, crypt depth, and villus/crypt ratio in duodenum, jejunum, and ileum. **(A)** The villus height, crypt depth, and villus/crypt ratio in the duodenum. **(B)** The villus height, crypt depth, and villus/crypt ratio in the jejunum. **(C)** The villus height, crypt depth, and villus/crypt ratio in the ileum. Statistical analysis was performed using one-way ANOVA followed by the LSD. *p < 0.05, **p < 0.01, ***p < 0.001.

In the histological study, no obvious pathological lesions were observed in the treatment groups, characterized by a regular, complete, and tightly arranged configuration of intestinal villi and crypts, along with an intact mucosal structure (Figure 4). The addition of WGHPE did not induce obvious pathological damage to the intestinal tract of laying hens. Figure 4 presents cross-sectional views of the duodenum, jejunum, and ileum, respectively. Structurally, no significant lesions were observed among the various treatment groups. As illustrated in Figure 5, the supplementation of different levels of WGHPE significantly enhanced both villus height and the V/C ratio in the jejunum and ileum (Figures 5C,D; *p* < 0.01), exhibiting a dose-dependent relationship; however, it had no significant effect on crypt depth (Figures 5C,D; *p* > 0.05). Notably, WGHPEM supplementation significantly increased duodenal crypt depth compared to the FLHS group (*p* < 0.05), yet neither villus height nor the V/C ratio was affected (*p* > 0.05).

### 3.5 Cecal microbiota

**Figure 6:**
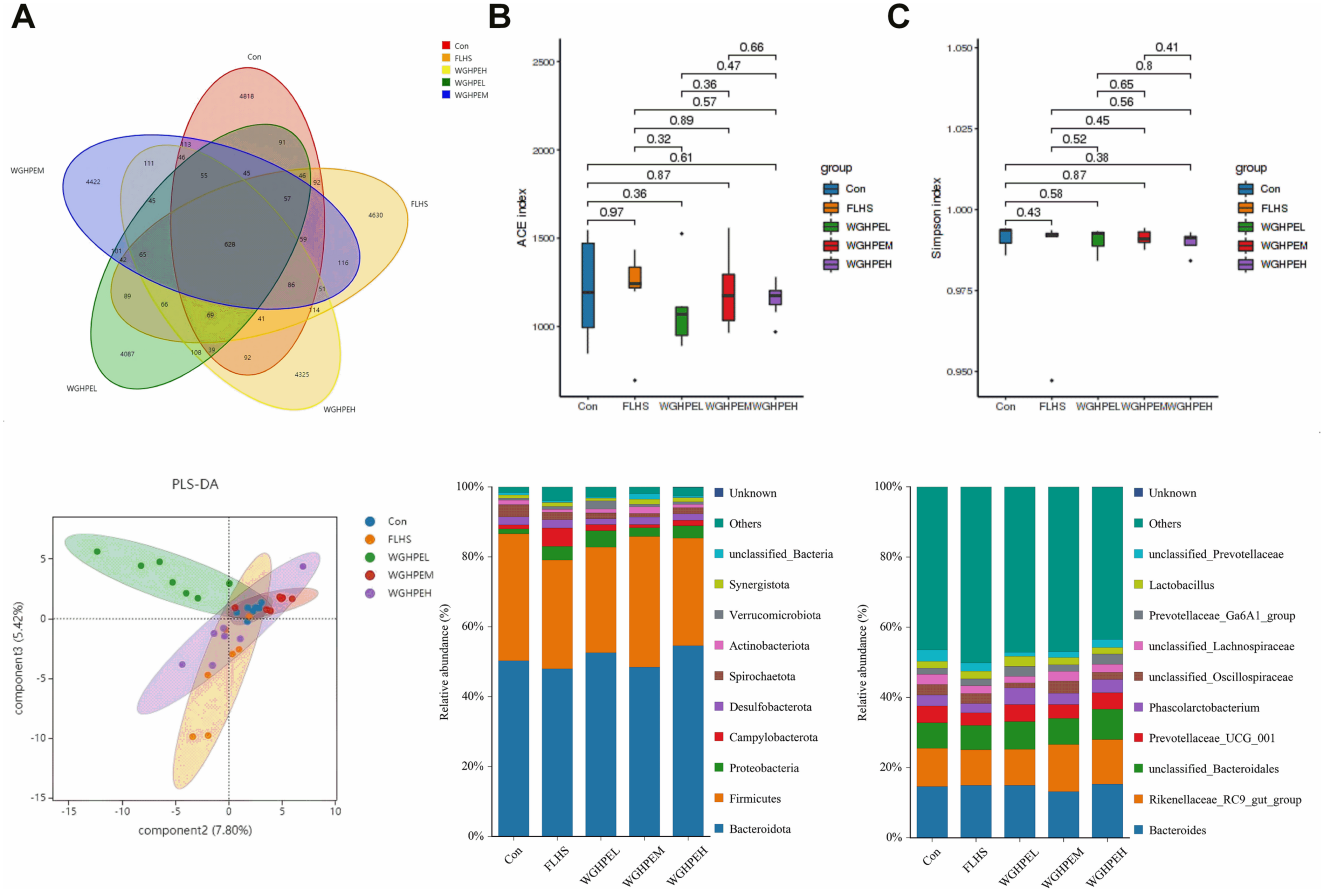
Walnut green husk polyphenol extracts (WGHPE) improved the gut microbial structure and taxonomic composition of induced-fatty liver hemorrhagic syndrome (FLHS) laying hens. **(A)** Venn diagram of operational taxonomic units (OTUs) in different groups. **(B)** The alpha diversity was evaluated by the Ace index. **(C)** The alpha diversity was evaluated by the Simpson index. **(D)** Partial least squares discriminant analysis (PLS-DA) diagram. Each point represents a sample; the points with the same color belong to the same group, and the points of the same group are marked by ellipses. **(E)** Community abundance of the cecal microflora community at the phylum level. **(F)** Community abundance of the cecal microflora community at the genus level.

The average sequencing depth was 48,977 ± 9,597 high-quality reads per sample, and rarefaction curves approached saturation, indicating adequate coverage. The Venn diagram illustrates that a total of 628 OTUs were shared across the five groups. Specifically, the Con, FLHS, WGHPEL, WGHPEM, and WGHPEH groups exhibited 4,818, 4,630, 4,078, 4,422, and 4,325 unique OTUs prior to rarefaction, respectively (Figure 6A). Analysis revealed that WGHPE did not significantly affect the *α*-diversity of the cecal microbiota (*p* > 0.05), indicating no significant changes in microbial richness or diversity (Figures 6B,C). For *β*-diversity assessment, partial least squares discriminant analysis (PLS-DA) was conducted. The findings indicated distinct clustering characteristics among microbial community structures across each group (Figure 6D).

The top 10 phyla and genera were presented in Figures 6E,F, respectively. Compared to the Con group, there was a decrease in the relative abundance of Firmicutes and Actinobacteriota in the FLHS group, while a marked increase in Proteobacteria and Campylobacterota was observed. Notably, treatment with WGHPE significantly ameliorated the effects of FLHS on each phylum (Supplementary Figure 1). Based on the inter-group difference analysis at the genus level, relative abundances of *Rikenellaceae_RC9_gut_group*, *Prevotellaceae_UCG_001*, *Phascolarctobacterium*, and *unclassified_Lachnospiraceae* were relatively low within the FLHS group; conversely, beneficial bacteria such as *Rikenellaceae_RC9_gut_group* and *Phascolarctobacterium* exhibited increased relative abundances in the WGHPE group (Figure 6F). Although a dose-dependent relationship was not observed, a distinct trend of improvement was noted, suggesting that the WGHPE intervention is advantageous for enhancing the intestinal microbiota structure in laying hens.

**Figure 7:**
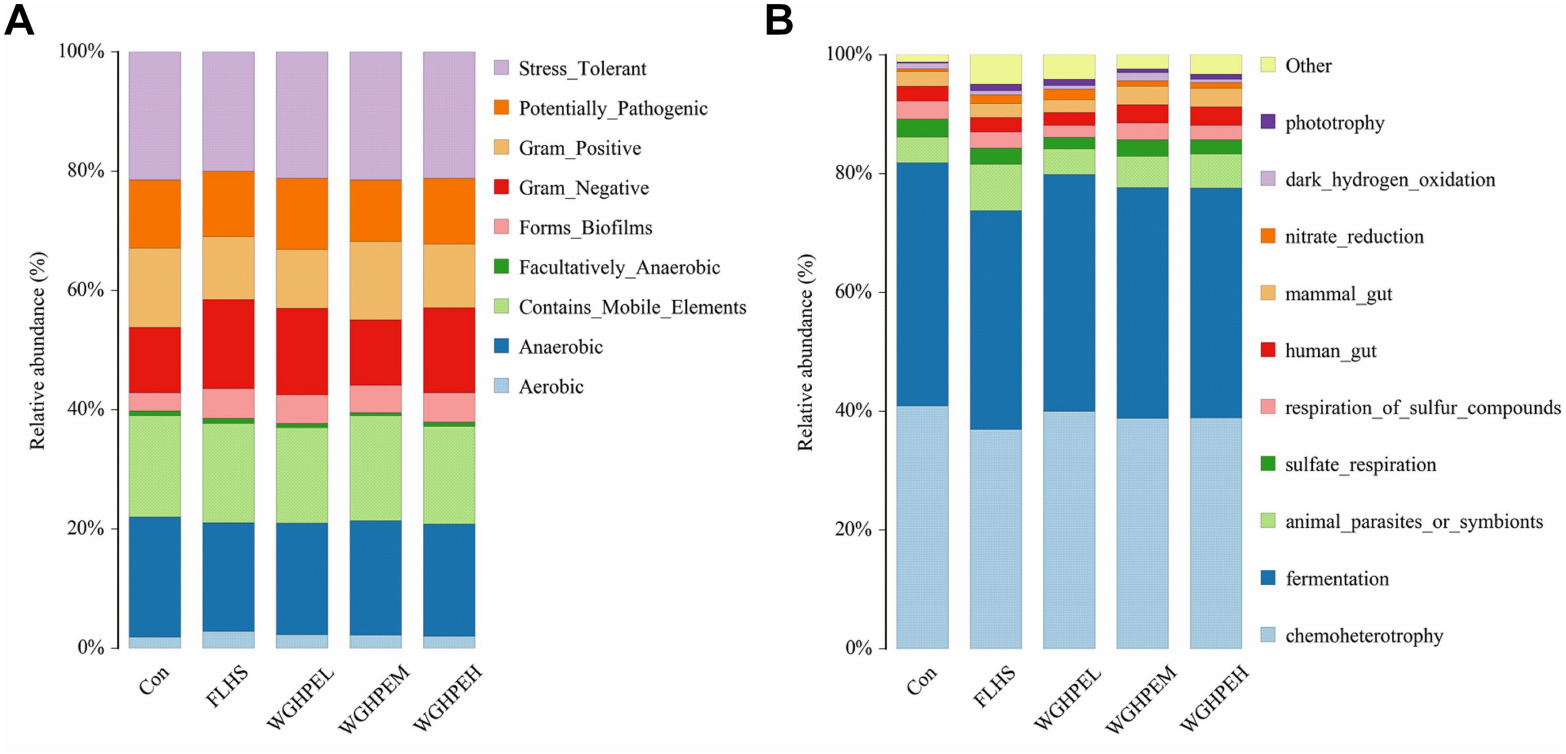
Functional gene prediction at the genus level of cecal microbiota in fatty liver hemorrhagic syndrome (FLHS) laying hens in response to dietary walnut green husk polyphenol extracts (WGHPE) supplementation. **(A)** The BugBase phenotypic prediction of the cecal microbiota at the genus level. **(B)** FAPROTAX functional prediction analysis of cecal microbiota at the genus level.

In the functional prediction analysis, the functional categories related to disease pathways showed significant differences (Supplementary Tables 3, 4). The BugBase algorithm was employed to normalize the features based on the predicted 16S copy number and to classify phenotypic information. WGHPEM has the potential to significantly reverse the elevated abundance of Gram-negative bacteria observed in the FLHS group, whereas the levels of Gram-positive bacteria exhibited a contrasting trend (*p* < 0.05; Figure 7A; Supplementary Tables 5, 6). The FAPROTAX algorithm was employed to predict the biochemical cycling processes occurring in the gut (34). Among these categories, microbial communities engaged in chemoheterotrophy and fermentation were found to be the most abundant, collectively exceeding a relative abundance of 70%. Although their presence in the intestinal microbiota of the FLHS group was relatively low, it showed an increasing trend (*p* > 0.05) under the influence of WGHPE (Figure 7B). The bacterial communities associated with fermentation, primarily comprising members from the *Delta-proteobacteria* class, *Enterobacterales* order, and *Actinomycetes* class, were notably enriched in the WGHPE treatment group (*p* > 0.05). Furthermore, the relative abundance of “animal parasites or symbionts” was markedly higher in the FLHS group compared to that in the Con group; however, this abundance decreased following WGHPE treatment and exhibited a trend towards normalization. Thus, incorporating WGHPE into dietary regimens can effectively regulate metabolic functions to support recovery within the intestinal microbial ecosystem.

**Figure 8:**
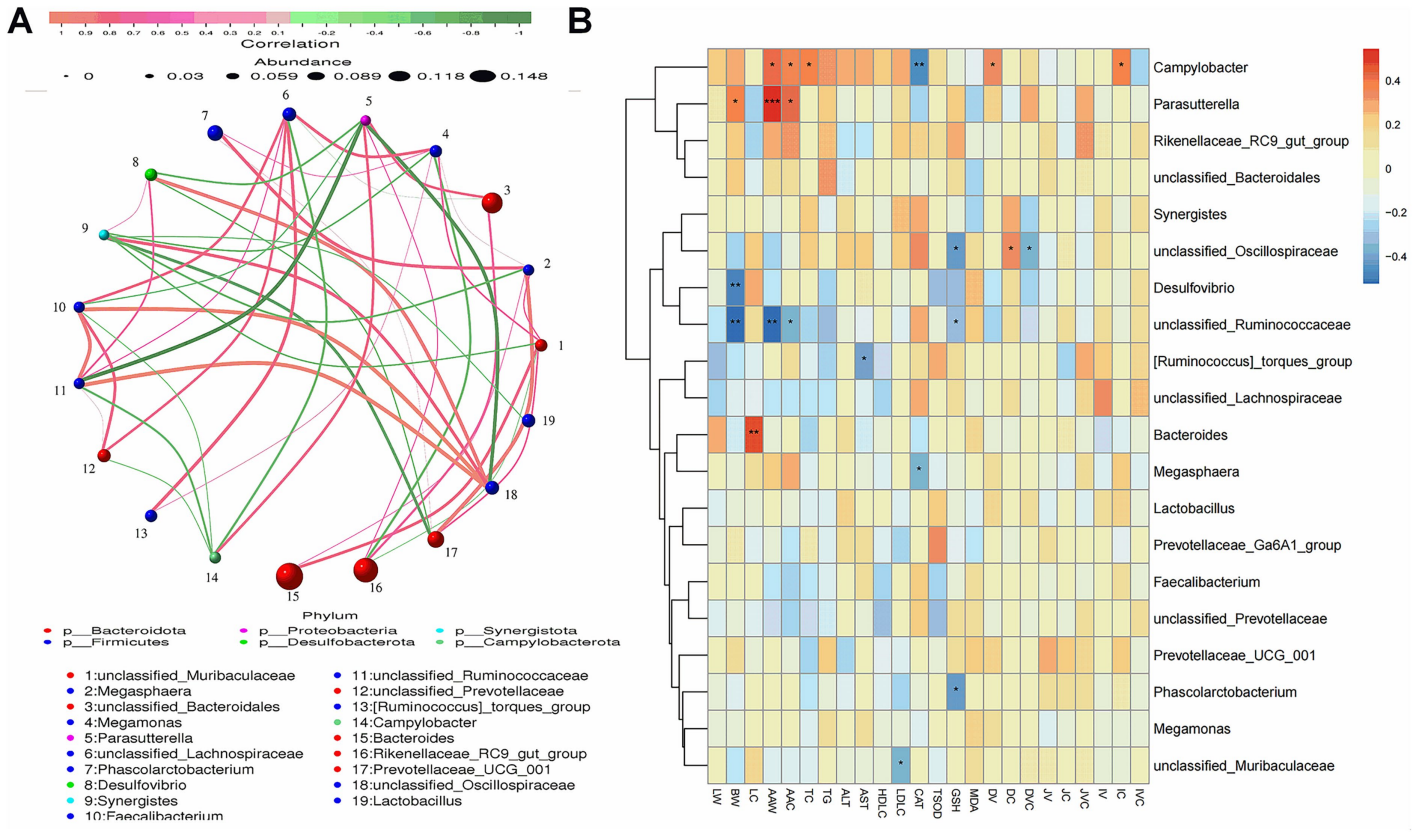
Correlation and association analysis of intestinal microbiota and indicators in fatty liver hemorrhagic syndrome (FLHS) laying hens in response to dietary walnut green husk polyphenol extracts (WGHPE) supplementation. **(A)** Correlation network diagram of intestinal microbiota and indicators in FLHS laying hens under the influence of WGHPE. Spherical nodes represent different species, with the size of each sphere indicating its abundance and the color denoting the various phyla to which these species belong. Lines illustrate the correlations between pairs of species, with line thickness reflecting the strength of these correlations. The colors of the lines are indicative of correlation type: red signifies a positive correlation, while green denotes a negative correlation. **(B)** Correlation network heatmap of intestinal microbiota and indicators in FLHS laying hens under the influence of WGHPE. Spearman correlation between intestinal microbiota and the markers of physiological, antioxidant, and histological morphology indicators. The intensity of the colors represents the degree of association (red, positive correlation; blue, negative correlation). LW, Liver weight; BW, Body weight; LC, Liver coefficient; AAW, Abdominal adipose weight; AAC, Abdominal adipose coefficient; TC, Total cholesterol; TG, Triglyceride; ALT, Alanine aminotransferase; AST, aspartate aminotransferase; HDLC, High-density lipoprotein cholesterol; LDLC, Low-density lipoprotein cholesterol; CAT, Catalase; TSOD, Total superoxide dismutase; GSH, Glutathione peroxidase; MDA, Malondialdehyde; DV, Duodenum villus height; DC, Duodenum crypt depth; DVC, Duodenum villus/crypt ratio; JV, Jejunum villus height; JC, Jejunum crypt depth; JVC, Jejunum villus/crypt ratio; IV, Ileum villus height; IC, Ileum crypt depth; IVC, Ileum villus/crypt ratio. *n* = 7 hens per group. Statistical analysis was performed using Spearman. *p < 0.05, **p < 0.01, ***p < 0.001.

A genus-level network Spearman correlation analysis of the gut microbiota among different groups of laying hens revealed that Megasphaera, *unclassified_Lachnospiraceae*, *Faecalibacterium*, *unclassified_Prevotellaceae*, *[Ruminococcus]_torques_group*, *Bacteroides*, and *unclassified_Oscillospiraceae* exhibited a strong positive correlation with other microorganisms. In contrast, *Parasutterella*, *Synergistes*, and *Campylobacter* demonstrated the most pronounced negative correlations. Additionally, *unclassified_Bacteroidales*, *Bacteroides*, and *Rikenellaceae_RC9_gut_group* displayed the highest relative abundance (Figure 8A). Correlation analysis between the intestinal microbiota and various physiological indicators (including antioxidant markers) in FLHS laying hens revealed that *Campylobacter* and *Pseudomonas* exhibited a highly significant positive correlation with body weight, abdominal adipose weight, and the abdominal adipose coefficient (*p* < 0.05). *Bacteroides* showed a significant positive correlation with the liver coefficient. In contrast, *Desulfovibrio* and *unclassified_Oscillospiraceae* were significantly negatively correlated with body weight, abdominal adipose weight, and abdominal adipose coefficient of FLHS laying hens (*p* < 0.05). Furthermore, *Campylobacter*, *unclassified_Oscillospiraceae*, *unclassified-Ruminococcaceae*, *Megasphaera*, and *Phascolarctobacterium* demonstrated significant negative correlations with antioxidant indices CAT and GSH-Px (*p* < 0.05). Notably, *Campylobacter* was significantly positively correlated with duodenal villi height and intestinal mucosa crypt depth; meanwhile, *unclassified_Oscillospiraceae* displayed a positive correlation with crypt depth of the duodenum but a negative correlation with the ratio of duodenal V/C value (Figure 8B).

## 4 Discussion

Nutrient demands increase sharply when hens enter the laying period. However, continuous high-energy intake can lead to lipid accumulation, intestinal ecological imbalance, and may even result in FLHS (35, 36). In the present study, the estrogen/corn oil induction method successfully recapitulated the key features of FLHS in laying hens. Compared with the control group, FLHS hens exhibited a 63.37% increase in abdominal fat content and a 26.51% elevation in liver weight (Figures 1B–E). Histopathologically, the FLHS model group showed expansion of cytoplasmic vacuoles in hepatocytes (Figure 1G), along with marked lipid droplet accumulation (Figure 1H) (37). These changes reflect sustained systemic and hepatic lipid metabolic disturbances induced by exogenous estrogen, which promotes hepatic *de novo* lipogenesis, leading to steatosis and hepatocellular ballooning. Unlike conventional FLHS models that rely on high-energy, low-protein diets, our induction method—using estrogen and corn oil without dietary manipulation—closely mimics the clinical signs, hemorrhage scores, and histopathological alterations described by Shini et al. (38), thereby better recapitulating naturally occurring FLHS. Previous studies reported that short-term estrogen followed by ad libitum feeding induces irreversible liver damage in approximately 88% of Hy-Line Brown hens. Importantly, our non-restrictive dietary modeling not only reduced hemorrhagic mortality but also preserved production and egg-laying performance (26), providing a more practical and humane experimental platform for evaluating interventions such as WGHPE.

Dietary supplementation with WGHPE, particularly at the 1.5% level, significantly alleviated FLHS-induced lipid metabolism disturbances. WGHPE treatment dose-dependently reduced serum TC, LDL-C, ALT, and AST levels in FLHS hens (Figure 2). These biochemical improvements were accompanied by reduced liver weight, abdominal fat deposition, and attenuation of vacuolar degeneration and lipid droplet accumulation (Figures 1F–H). The WGHPE used in this study contained 10 identified polyphenolic compounds (Supplementary Table 1). Among these, rutin (39, 40), Coumalic acid (41), Caffeic acid (42) and quercetin (43) are known to inhibit hepatic lipogenesis and activate Nrf2-mediated antioxidant responses; chlorogenic acid (44, 45) has been shown to reduce serum ALT and AST levels and alleviate hepatic steatosis in laying hens; and hyperoside has been shown to significantly reduce serum and liver TC and TG, as well as serum LDL-C levels, in hyperlipidemic mice (46). Ferulic acid (47), coumalic acid (41), and neohesperidin dihydrochalcone (48) possess strong antioxidant and anti-inflammatory properties. Thus, the observed hepatoprotective and antioxidant effects of WGHPE are likely attributable to the synergistic actions of these multiple polyphenolic constituents. Consistent with our findings, Zhang et al. reported that walnut green husk extract lowered TC, TG, and LDL-C in acutely hyperlipidemic mice (49). Collectively, these observations highlight the consistent hepatoprotective and lipid-lowering properties of walnut-derived extracts. The multi-component synergy of various polyphenols, rather than a single molecule, likely mediates these beneficial effects, supporting the potential application of WGHPE in managing metabolic disorders such as fatty liver hemorrhagic syndrome in poultry.

Oxidative stress is a well-established etiological driver of FLHS. In our study, FLHS induction significantly decreased serum CAT and T-SOD activities, while increasing MDA levels—a stable end-product of lipid peroxidation (Figure 3). Treatment with WGHPE markedly elevated CAT and T-SOD activities and reduced MDA levels in a dose-dependent manner. These findings indicate that WGHPE augments systemic antioxidant capacity and effectively inhibits lipid peroxidation *in vivo*, likely involving activation of the Nrf2/ARE signaling pathway, which upregulates the expression of endogenous antioxidant enzymes. In contrast, serum GSH-Px activity remained unchanged across groups, which may reflect that CAT and T-SOD are more sensitive to the oxidative challenge in this FLHS model. Furthermore, the triglyceride-to-HDL-cholesterol (TG/HDL-C) ratio, a surrogate marker of insulin sensitivity, was improved by WGHPE, suggesting that the extract may alleviate hepatic steatosis partly by ameliorating insulin resistance, thereby reducing circulating lipid substrate availability and *de novo* lipogenesis (50–52). Our results align with previous reports that polyphenol-rich extracts enhance antioxidant enzyme activities and reduce oxidative damage in various models (53–55). Squires and Wu (56) also reported significantly elevated hepatic MDA concentrations in hens with severe liver hemorrhage, consistent with our observations. This mechanism may help mitigate oxidative damage induced by external stressors, reduce insulin resistance associated with oxidative stress, and lower the incidence of FLHS in laying hens (57–59), highlighting the potential of WGHPE as an antioxidant-based nutritional strategy.

Intestinal barrier dysfunction is closely linked to the pathogenesis of chronic liver diseases (60). In this study, WGHPE supplementation significantly increased villus height and the V/C ratio in the jejunum and ileum of FLHS hens (Figure 5), indicating improved nutrient digestion and absorption. Notably, no obvious pathological damage to the intestinal tract was observed, and the effect was dose-dependent. These morphological improvements suggest that WGHPE may enhance intestinal barrier integrity, potentially by reducing intestinal permeability, limiting endotoxin translocation, and thereby attenuating hepatic inflammation. Consistent with the findings of Wang et al., dietary supplementation with the ethyl acetate extract derived from walnut green husk similarly elevated VH and V/C ratios in the jejunum and ileum of broilers (11). Moreover, dietary or drinking-water supplementation with polyphenol-rich plant extracts—including rutin (40), ferulic acid (61), and grape proanthocyanidins (62)—has been demonstrated to restore intestinal epithelial architecture and improve intestinal health in broilers. Furthermore, in a murine model of high-fat diet-induced colonic injury, walnut green husk polysaccharides ameliorated intestinal microbiota dysbiosis (63). By enhancing mucosal morphology, WGHPE likely preserves intestinal barrier function, which is a critical component of the gut-liver axis and represents a key target for preventing FLHS progression.

The gut microbiota plays a critical role in the pathogenesis of FLHS. Our intervention with WGHPE successfully reduced cecal abundance of the pathogenic bacterium *Campylobacter* and increased the relative abundance of beneficial bacterial taxa, including *[Ruminococcus]_torques_group*, *Desulfovibrio*, and *unclassified_Ruminococcaceae* (Figure 6F). *Rikenellaceae_RC9_gut_group* and *Phascolarctobacterium* are dominant cecal genera in laying hens that ferment fiber to produce propionate, a short-chain fatty acid known to inhibit hepatic lipogenesis and reduce feed intake (43, 64). Supporting their beneficial role, dietary supplementation with daidzein (a phenolic compound) has been reported to increase the abundance of *Rikenellaceae_RC9_gut_group* in late-laying hens, accompanied by improved performance and antioxidant capacity (65). Functional prediction using BugBase revealed that WGHPE reversed the elevated abundance of Gram-negative bacteria observed in the FLHS group (Figure 7A). But this remains speculative given the cross-sectional nature of the data. Furthermore, WGHPE may exert direct antimicrobial effects against *Campylobacter*, which could also explain the reduced abundance (11). Correlation analysis further demonstrated that *Campylobacter* abundance was significantly negatively correlated with CAT activity, suggesting that WGHPE may suppress *Campylobacter* proliferation by upregulating host antioxidant enzyme activity, thereby protecting intestinal epithelial cells from oxidative damage and creating an unfavorable niche for this pathogen. Prior clinical and preclinical studies have associated higher abundances of *[Ruminococcus]_torques_group* and *Desulfovibrio* with a reduced risk of alcoholic liver disease (66). Consistent with findings reported by Wu et al. (67), our study also revealed a significant negative correlation between the abundance of *[Ruminococcus]_torques_group* and serum AST levels. Previous studies have demonstrated that walnut green husk extract promotes intestinal colonization by beneficial bacteria, enhances mucosal immunity, and ameliorates gut microbiota dysbiosis (63, 68). These findings suggest that WGHPE modulates the cecal microbiota in a manner that reduces hepatic lipid accumulation and inflammation, supporting the gut-liver axis as a therapeutic target for FLHS. However, these cecal correlations are cross-sectional and do not imply direct causation.

Although this study provides compelling evidence that WGHPE alleviates liver injury, ameliorates lipid metabolism disorders, and restores intestinal homeostasis in the FLHS model of laying hens, several limitations should be acknowledged. First, our study is primarily descriptive and correlational; thus, the causal relationships between specific microbial taxa and the observed physiological improvements remain to be established. Second, the control group did not receive a sham injection of the corn oil vehicle, meaning that we cannot entirely exclude the potential influence of the injection procedure or the oil itself on baseline outcomes. Nevertheless, the marked contrast between the Con and FLHS groups, together with the dose-dependent amelioration observed following WGHPE supplementation, strongly suggests that the major effects are attributable to *β*-estradiol induction and the dietary intervention. Third, functional predictions generated using PICRUSt2, BugBase, and FAPROTAX are inferential and rely on databases heavily biased toward mammalian (especially human) gut microbiomes, with limited representation of poultry-specific genomes. Therefore, these predictions should be interpreted as exploratory; direct metagenomic sequencing or metabolomics is needed to confirm inferred functional changes such as enhanced fermentation capacity. In future work, we will integrate untargeted metabolomics with FMT to elucidate whether WGHPE-modulated gut microbiota are sufficient to confer protection against FLHS. Addressing these limitations will provide a more comprehensive understanding of how WGHPE exerts its hepatoprotective effects and will facilitate the development of WGHPE as a plant-derived feed additive for laying hens.

## 5 Conclusion

The incorporation of WGHPE into the diets of FLHS laying hens exerts multiple beneficial functions, including reducing oxidative stress, improving intestinal health, decreasing fat deposition and blood lipid levels, and alleviating FLHS-associated liver injury. These results may offer a novel nutritional strategy for mitigating liver damage in laying hens caused by FLHS. Furthermore, the association network between the intestinal microbiota and host physiological indicators within the FLHS model establishes a foundation for further elucidating the relationship between intestinal microbiota and host physiology. Future research should employ multi-omics analysis (including metabolomics) to explore the effects of WGHPE on the intestinal microbiota.

## Data Availability

The datasets presented in this study can be found in online repositories. The names of the repository/repositories and accession number(s) can be found at: https://www.ncbi.nlm.nih.gov/, PRJNA1418150.

## Footnotes

1
https://www.hyline.com/literature/brown

2
https://www.biocloud.net

## Supplementary material

SUPPLEMENTARY FIGURE 1 Relative abundance of representative cecal bacterial taxa at selected genus-like levels in laying hens with fatty liver hemorrhagic syndrome (FLHS) following dietary supplementation with walnut green husk polyphenol extract (WGHPE). Bars represent mean relative abundance ± SEM across experimental groups. Statistical differences were analyzed using one-way analysis of variance (ANOVA) followed by the least significant difference (LSD) *post-hoc* test. Significance levels are indicated as **p* < 0.05, ***p* < 0.01, and ****p* < 0.001.
