# Supplementary material for: Dietary supplementation with walnut (Juglans regia L.) green husk polyphenol extract mitigates fatty liver hemorrhagic syndrome in laying hens
Source: Front Vet Sci. 2026 Jun 12;13:1803328. doi: 10.3389/fvets.2026.1803328 (PMC13308526; doi:10.3389/fvets.2026.1803328)
Supplement: Supplementary file 2 [file Table_1.docx]

**Table S1. High‑performance liquid chromatography (HPLC) analysis of the principal phenolic compounds in walnut green husk polyphenol extract.**

| Retention time (min) | Compound | CAS Number | Concentration (mg/mL) | MW | MF | Structure |
| --- | --- | --- | --- | --- | --- | --- |
| 21.10 | Coumalic acid | 500-05-0 | 1.29 | 140.09 | C_6_H_4_O_4_ | 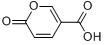 |
| 36.97 | 1. Hydroxybenzoic acid | 99-96-7 | 0.20 | 138.12 | C_7_H_6_O_3_ | 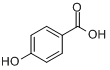 |
| 37.40 | Chlorogenic acid | 327-97-9 | 1.00 | 354.31 | C_16_H_18_O_9_ | 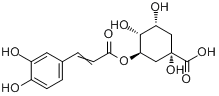 |
| 44.99 | Caffeic acid | 331-39-5 | 1.16 | 180.16 | C_9_H_8_O_4_ | 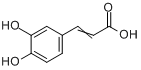 |
| 56.97 | Rutin | 153-18-4 | 2.02 | 664.57 | C_27_H_30_O_16_·3H_2_O | 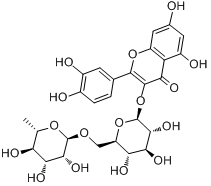 |
| 63.12 | Hyperoside | 482-36-0 | 1.00 | 464.38 | C_21_H_20_O_12_ | 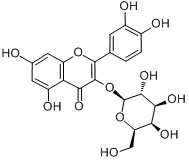 |
| 71.77 | Taxifolin | 480-18-2 | 0.20 | 304.25 | C_15_H_12_O_7_ | 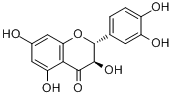 |
| 77.79 | Ferulic acid | 1135-24-6 | 0.20 | 194.18 | C_10_H_10_O_4_ | 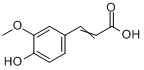 |
| 111.54 | Neohesperidin dihydrochalcone | 20702-77-6 | 1.00 | 612.58 | C_28_H_36_O_15_ | 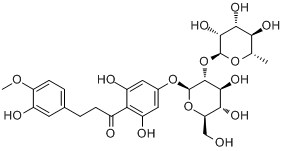 |
| 121.04 | Quercetin | 117-39-5 | 2.00 | 302.24 | C_15_H_10_O_7_ | 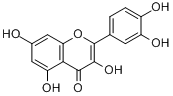 |

Notes: Precisely 10 µL aliquots of the test sample solution and the individual reference standard solutions (coumalic acid, 4-hydroxybenzoic acid, rutin, neohesperidin dihydrochalcone, taxifolin, ferulic acid, chlorogenic acid, caffeic acid, quercetin and hyperoside) were separately injected into an Agilent 1260 high-performance liquid chromatography (HPLC) system. Chromatographic separation was performed on a Diamonsil C_18_ column (250 mm × 4.6 mm, 5 µm particle size) packed with octadecylsilane-bonded silica gel. The mobile phase consisted of acetonitrile and 0.2% (v/v) phosphoric acid aqueous solution, and gradient elution was employed. Detection wavelengths were set as follows: 254 nm for 4-hydroxybenzoic acid and coumalic acid; 275 nm for rutin; 283 nm for neohesperidin dihydrochalcone; 290 nm for taxifolin; 316 nm for ferulic acid; 326 nm for chlorogenic acid and caffeic acid; 360 nm for quercetin and hyperoside. Abbreviations: MW, molecular weight; MF, molecular formula.

**Table S2. Ingredients and nutrient composition of the experimental diets fed to laying hens.**

| Composition (%) | | Nutrient levels** | |
| --- | --- | --- | --- |
| Corn | 65.00 | Metabolizable energy (MJ/kg) | 11.04 |
| Soybean meal | 20.00 | Crude protein (%) | 16.05 |
| Cottonseed meal | 3.00 | Ca (%) | 3.65 |
| Wheat bran | 1.65 | Available P (%) | 0.49 |
| CaHPO_4_ | 1.00 | Met+Cys (%) | 0.58 |
| Dicalcium phosphate | 0.35 | Lys (%) | 0.73 |
| Limestone | 8.00 | Met (%) | 0.33 |
| *Premix | 1.00 |  |  |
| Total | 100.00 |  |  |

* Premix provides (per kg of the diet): vitamin A 330,000 IU, vitamin D_3_ 82,500 IU, vitamin E 2,000 IU, vitamin K_3_ 180 mg, vitamin B_1_ 120 mg, vitamin B_2_ 500 mg, vitamin B_6_ 300 mg, vitamin B_12_ 1.2 mg, nicotinic acid 2.4 g, calcium pantothenate 1.2 g, folic acid 60 mg, biotin 15 mg, copper 0.8 g, iron 6 g, zinc 6 g, manganese 9 g, selenium 21 mg, iodine 90 mg.

** Nutrient levels are calculated values; ingredient percentages are as‑formulated.

**Table S3. Predicted functional profiles of the cecal microbiota based on PICRUSt2 analysis in laying hens from the control (Con) group compared with those from the fatty liver hemorrhagic syndrome (FLHS) model group (Con vs. FLHS).**

| Class | Con  rel.freq. (%) | Con  std.dev. (%) | FLHS  rel.freq. (%) | FLHS  std.dev. (%) | P value |
| --- | --- | --- | --- | --- | --- |
| Metabolism | 79.368 | 0.334 | 79.225 | 0.434 | 0.533 |
| Genetic information processing | 8.749 | 0.125 | 8.694 | 0.066 | 0.367 |
| Environmental information processing | 5.424 | 0.237 | 5.469 | 0.237 | 0.746 |
| Cellular processes | 2.750 | 0.135 | 2.844 | 0.241 | 0.422 |
| Human diseases | 2.329 | 0.019 | 2.391 | 0.057 | 0.037 |
| Organismal systems | 1.380 | 0.034 | 1.376 | 0.026 | 0.811 |

**Table S4. Predicted functional profiles of the cecal microbiota based on PICRUSt2 analysis in laying hens from the fatty liver hemorrhagic syndrome (FLHS) model group compared with those supplemented with medium‑dose walnut green husk polyphenol extract (WGHPEM) (FLHS vs. WGHPEM).**

| Class | WGHPEM  rel.freq. (%) | WGHPEM  std.dev. (%) | FLHS  rel.freq. (%) | FLHS  std.dev. (%) | P value |
| --- | --- | --- | --- | --- | --- |
| Metabolism | 79.353 | 0.165 | 79.225 | 0.434 | 0.517 |
| Genetic information processing | 8.769 | 0.078 | 8.694 | 0.066 | 0.099 |
| Environmental information processing | 5.431 | 0.100 | 5.469 | 0.237 | 0.723 |
| Cellular processes | 2.751 | 0.091 | 2.844 | 0.241 | 0.402 |
| Human diseases | 2.325 | 0.029 | 2.391 | 0.057 | 0.034 |
| Organismal systems | 1.370 | 0.019 | 1.376 | 0.026 | 0.652 |

**Table S5. Predicted phenotypic traits of the cecal microbiota based on BugBase analysis in laying hens from the control (Con) group compared with those from the fatty liver hemorrhagic syndrome (FLHS) model group (Con vs. FLHS).**

| Phenotype | Con  rel.freq. (%) | Con  std.dev. (%) | FLHS  rel.freq. (%) | FLHS  std.dev. (%) | P value |
| --- | --- | --- | --- | --- | --- |
| Aerobic | 7.829 | 2.624 | 11.009 | 6.874 | 0.322 |
| Anaerobic | 83.113 | 4.465 | 72.482 | 12.734 | 0.092 |
| Contains mobile elements | 70.322 | 4.581 | 65.996 | 7.081 | 0.237 |
| Facultatively anaerobic | 3.309 | 0.965 | 3.138 | 1.827 | 0.844 |
| Forms biofilms | 12.634 | 1.487 | 19.072 | 9.989 | 0.167 |
| Gram-negative | 45.185 | 5.894 | 57.695 | 11.962 | 0.048 |
| Gram-positive | 54.816 | 5.894 | 42.305 | 11.962 | 0.048 |
| Potentially pathogenic | 47.550 | 4.862 | 44.149 | 11.197 | 0.514 |
| Stress tolerant | 88.739 | 3.838 | 79.776 | 15.769 | 0.220 |

**Table S6.** **Predicted phenotypic traits of the cecal microbiota based on BugBase analysis in laying hens from the fatty liver hemorrhagic syndrome (FLHS) model group compared with those receiving medium‑dose walnut green husk polyphenol extract (WGHPEM)** **(FLHS vs. WGHPEM).**

| Phenotype | FLHS  rel.freq. (%) | FLHS  std.dev. (%) | WGHPEM  rel.freq. (%) | WGHPEM  std.dev. (%) | P value |
| --- | --- | --- | --- | --- | --- |
| Aerobic | 11.009 | 6.874 | 9.169 | 2.454 | 0.555 |
| Anaerobic | 72.482 | 12.734 | 79.668 | 4.645 | 0.232 |
| Contains mobile elements | 65.996 | 7.081 | 73.034 | 3.604 | 0.058 |
| Facultatively anaerobic | 3.138 | 1.827 | 2.251 | 0.593 | 0.294 |
| Forms biofilms | 19.072 | 9.989 | 18.780 | 3.200 | 0.947 |
| Gram-negative | 57.695 | 11.962 | 45.439 | 4.398 | 0.048 |
| Gram-positive | 42.305 | 11.962 | 54.561 | 4.398 | 0.048 |
| Potentially pathogenic | 44.149 | 11.197 | 43.004 | 2.601 | 0.815 |
| Stress tolerant | 79.776 | 15.769 | 89.045 | 2.698 | 0.203 |
